# CGRP Regulates Nucleus Pulposus Cell Apoptosis and Inflammation via the MAPK/NF-*κ*B Signaling Pathways during Intervertebral Disc Degeneration

**DOI:** 10.1155/2021/2958584

**Published:** 2021-12-26

**Authors:** Kaiqiang Sun, Jian Zhu, Chen Yan, Fudong Li, Fanqi Kong, Jingchuan Sun, Xiaofei Sun, Jiangang Shi, Yuan Wang

**Affiliations:** Department of Orthopedic Surgery, Changzheng Hospital, Navy Medical University, No. 415 Fengyang Road, Shanghai 200003, China

## Abstract

Chronic low back pain (CLBP) has been proved to be the dominating cause of disability in patients with lumbar degenerative diseases. Of the various etiological factors, intervertebral disc degeneration (IVDD) has been the dominating cause. In the past few decades, the role and changes of nerve systems, especially the peripheral sensory fibers and their neurotransmitters, in the induction and progression of IVDD have attracted growing concerns. The expression of many neuropeptides, such as SP, NPY, and CGRP, in the nociceptive pathways is increased during the progression of IVDD and responsible for the discogenic pain. Here, the role of CGRP in the progression of IVDD was firstly investigated both in vitro and in vivo. Firstly, we confirmed that human degenerated intervertebral disc tissue exhibited elevated expression of CGRP and its receptor. Secondly, in vitro experiments suggested that CGRP could inhibit the proliferation and induce apoptosis in human nucleus pulposus (NP) cells, as well as promote inflammation and degenerated phenotypes through activating NF-*κ*B and MAPK signaling pathways. Thirdly, CGRP receptor antagonist, Rimegepant, can ameliorate the adverse effects of CGRP imposed on NP cells, which were confirmed in vitro and in vivo. Our results will bring about a brand-new insight into the roles of neuromodulation in IVDD and related therapeutic attempts.

## 1. Introduction

Chronic low back pain (CLBP) has been the dominating reason for daily disability and repeating clinic visit in patients with lumbar degenerative diseases (LDDs) and brings tremendous social-economical-clinical impact worldwide [1–3]. Among the multiple etiological contributors, symptomatic intervertebral disc degeneration (IVDD) is acknowledged as the most common one. From an anatomical perspective, the intervertebral disc tissue is composed of three main parts that comes from different embryologic origins: the gelatinous nucleus pulposus tissue (NP), chondrocyte-like annulus fibrosus tissue (AF), and cartilaginous endplate tissue (CEP) [4, 5]. In general, IVDD, being an age-related biological process, possesses characteristics of reduced hydration and extracellular matrix (ECM), increasing ingrowth of neurovascular structures, and extensive release of inflammation-related cytokines within the NP tissue, which finally results in spinal instability and CLBP [6, 7]. Accumulating evidence has suggested that IVDD can be affected by multifactorial pathogenesis, including age, gender, repetitive mechanic load exposure, and hereditary factors [8–11]. However, the exact pathophysiological cause of IVDD remains to be interpreted in detail.

The normal structure of IVD is avascular that is poorly innervated, with the most nerve fibers existing in the outer 1/3 of AF [12]. In the past few decades, the role and changes of nerve systems during the progression of IVDD have attracted growing attention among researches [13]. A recent scope review suggested that during the process of IVDD, the neurovascularization within the disc, especially the NP tissue, is gradually evident, which is frequently located at regions of tissue granulation, tears, and lesions [14]. Notably, nerve fibers did not always accompany vasculature with degenerated IVD [14] Additionally, the abundance of nerve fibers increases with the progression of IVDD [15]. Among the never fibers, peripheral sensory fibers and their neurotransmitters have been the most noticed for their role in discogenic pain, such as neuropeptide Y (NPY), substance P (SP), and calcitonin gene-related peptide (CGRP) [16, 17]. Peripheral sensory fibers and nociceptors are highly sensitive to various noxious stimuli, including inflammatory, thermal, and mechanical stimuli. However, whether the role of these neurotransmitters in IVDD is beyond discogenic pain remains unclear, and few studies have been published investigating the direct relationship between these neurotransmitters and IVDD. It was reported recently that SP could directly promote the proinflammatory cytokine release in the intervertebral disc [18]. Conversely, in our previous study, NPY was found to prevent NP cells from IL-1*β*-mediated cell apoptosis and ECM degradation [19].

CGRP, a peptide with 37 amino acids, is expressed predominantly on sensory nerve fibers [20]. CGRP has two forms, *α* and *β* CGRP, and in the peripheral nervous system, *α* CGRP is the major form [20, 21]. The CGRP receptor is a complex composed of multiple proteins, and the two transmembrane proteins, calcitonin receptor-like receptor (CALCRL) and receptor activity-modifying protein 1 (RAMP1), are critical to CGRP-related effects [22–24]. In general, CALCRL, as the member of the secretin receptor family, is critical as receptors for both CGRP and adrenomedullin [20]. However, in order to exhibit function for CGRP, CALCRL must bind to RAMP1 to form a heterodimer. Thus, coexpression of CALCRL and RAMP1 is necessary for a cell to respond to CGRP [21]. A previous study demonstrated the important relationship between the level of CGRP expressed on osteoarthritis-related pain [25]. Stöckl et al. demonstrated that CGRP could induce senescence and apoptosis of chondrocytes and decreased the chondrogenic marker expressed during the progression of osteoarthritis [26]. Interestingly, increased expression of CGRP was found in degenerated IDD [27]. Many analogies between IVD and articular cartilage have been conducted since chondrocytes and NP cells share similar biological characteristics, which raises the question whether CGRP can directly participate in the process of IVDD.

Therefore, this current study was designed to elucidate (1) the expression profile of CGRP and its receptors in different severities of IVDD and aging IVD, (2) the direct bioeffects of CGRP on human NP cells and its pathological mechanisms, and (3) the preliminary therapeutic effect of CGRP receptor antagonist, Rimegepant, on IVDD in vivo. The results will bring about a brand-new insight into the roles of neuromodulation in IVDD and related therapeutic attempts.

## 2. Methods and Materials

### 2.1. Collection of Human Samples

Twenty-four patients (age ranging from 23 to 56 years old) undergoing surgical fusion in our institution were enrolled. Among them, 8 patients were diagnosed with spinal trauma and 16 were diagnosed with intervertebral disc herniation. NP tissues were harvested intraoperatively and divided into Grades II-V in reference to Pfirrmann grade system on preoperative magnetic resonance imaging (MRI). NP tissues from 20 patients were isolated and used to perform immunofluorescence and immunohistochemistry; NP tissues from 4 patients with Grade II were utilized to in vitro isolate NP cells for cell experiments.

The study was reviewed and approved by the ethics committee of our institution, and all patients consented to this project.

### 2.2. Isolating and Culturing Human NP Cells [19, 28]

Collected human IVD tissues were stored with the 0.9% sterile saline for further transportation to our laboratory. Once the tissue is obtained, we firstly washed the tissue using PBS (Servicebio, China) for 3 times. Subsequently, the gel-like NP tissue was isolated. Next, NP cells would be released using Trypsin-EDTA (Beyotime, Shanghai, China), followed by type II collagenase (100 mg, AC12L141, China). Following discarding the digestive fluid, the NP cells would be cultured at an incubator at 37°C and 5% CO2 for the following experiments.

### 2.3. Assay of Cell Viability

Human NP cells were stimulated with various doses of CGRP (10^−11^, 10^−10^, 10^−9^, 10^−8^, 10^−7^, 10^−6^, and 10^−5^ M) for 24 hours, followed by incubation with CCK 8 solution. The absorbance of 450 nm was then measured to visualize cell viability.

### 2.4. Quantitative Real-Time Polymerase Chain Reaction and Western Blot

These two methods have been depicted in detail in our previous study [28]. The primer sequences are summarized in Table 1. The primary antibodies in this study included CGRP (ab283568, 1 : 1000, Abcam, USA), CALCRL (861587, Zen Bio, Chengdu, China), RAMP1(385544, Zen Bio, Chengdu, China), Bax (200958, 1 : 1000, Zen Bio, Chengdu, China), Bcl2 (381702, 1 : 1000, Zen Bio, Chengdu, China), Cleaved-caspase3, iNOS (1 : 1000, Zen Bio, Chengdu, China), COX-2 (1 : 1000, Zen Bio, Chengdu, China), MMP3 (384995, 1 : 1000, Zen Bio, Chengdu, China), type I collagen (1 : 1000, ab34710, Abcam), type II collagen (1 : 1000, Zen Bio, Chengdu, China), aggrecan (ab36861, 1 *μ*g/mL, Abcam, USA), NF-*κ*B p65 (D14E12, #8242, Cell Signaling Technology, Inc., three Trask Lane Danvers, United States), p-p65 (Ser536; #3033, Cell Signaling Technology, Inc., three Trask Lane Danvers, United States), Ik-Ba (380682, 35 kDa; Zen Bio, Chengdu, China, 1 : 1,000), p-Ik-Ba (340776, 35 kDa; Zen Bio, Chengdu, China, 1 : 1,000), ERK1/2 (201245-4A4, 42/44 kDa; Zen Bio, Chengdu, China, 1 : 1,000), p-ERK1/2 (301245, 42/44 kDa; Zen Bio, Chengdu, China, 1 : 1,000), p38 (200782, 43 kDa; Zen Bio, Chengdu, China, 1 : 500), p-p38 (310069, 43 kDa; Zen Bio, Chengdu, China, 1 : 1,000), JNK (381100, 46/54 kDa; Zen Bio, Chengdu, China, 1 : 1,000), and p-JNK (380556, 46/54 kDa; Zen Bio, Chengdu, China, 1 : 1,000).

### 2.5. Assay of Cell Apoptosis [28]

Briefly, well-prepared human NP cells were prepared using Triton X-100, followed by adding terminal deoxynucleotidyl transferase dUTP nick-end labeling (TUNEL) reaction solution. Then, samples would be washed for three times. Subsequently, NP cells were stained with DAPI solution. Finally, a fluorescence microscope was used to detect the TUNEL-positive cells (570 nm, Olympus, Japan).

### 2.6. Immunohistochemical Analysis and Immunofluorescence Staining

These two methods have been explained in detail in our previous study [28]. The primary antibodies included CGRP (ab283568, 1 : 1000, Abcam, USA), CALCRL, RAMP1, type II collagen, Cleaved-caspase-3, iNOS, aggrecan, MMP3, and NF-*κ*B p65. The secondary antibody included HRP-conjugated secondary antibody for goat or mouse (GB23303, Servicebio), Cy3-conjugated Goat Anti-mouse IgG (GB21301, Servicebio), and FITC-conjugated Goat Anti-Rabbit IgG (GB22303, Servicebio).

### 2.7. Safranin O-Fast Green Staining

Briefly, the paraffin slides were dewaxed through pure xylene for 20-30 minutes, pure ethanol for 5-10 minutes, and 75% ethanol for another 5 minutes. The slices were then stained by fast green dye solution for 2 minutes and washed under tap water. Consequently, the slices were stained by Safranin O dye solution for 3-5 seconds and dehydrated rapidly in absolute ethanol for 5 seconds. A light microscope was used to capture the images finally (Olympus, Japan).

### 2.8. Establishment of Needle Injury-Induced IVDD Mouse Model and Treatment

Firstly, the mice were randomly divided into three groups (*n* = 5 per group): sham surgery group, injury group, and injury+Rimegepant (T4610, CAS 1289023-67-1, CGRP receptor antagonist, TOPSCIENCE, Shanghai, China) group. For establishment of IVDD in vivo, the anesthesia of mouse was induced by 3% isoflurane and maintained by 1.5% isoflurane under sterile conditions. The tail disc at Co 4/5 was punctured using sterilized 22-gauge needle (penetrated-rotated repeatedly held for 30 s). For IVDD treatment, Rimegepant was administrated orally (9 mg/kg/day) for 1 month before sacrificing, and the dose was calculated in reference to previous studies using the metrological conversion formula between human and mouse [29, 30]. The tail discs were harvested at 1 month postoperatively for following histochemical experiments.

The animal experiment in this present study has been approved by the review board of our institution.

## 3. Results

### 3.1. Elevated Expression of CGRP and Its Receptor in Degenerated Human IVD

**Figure 1:**
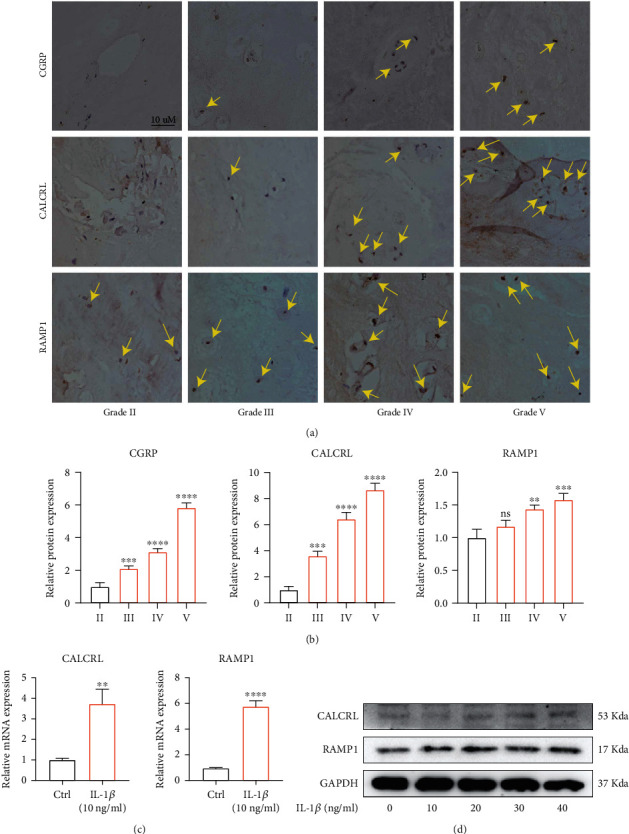
Elevated expression of CGRP and its receptor in degenerated human IVD tissue. (a, b) The protein expression of CGRP and its receptors, CALCRL and RAMP 1, was increased with the progression of IVDD. (c) Higher mRNA expression of CALCRL and RAMP1 was observed in IL-1*β*- (10 ng/mL) treated NP cells. (d) The protein amount of CGRP receptors, CALCRL and RAMP1, in human nucleus pulposus inn vitro was gradually elevated with the increasing concentration of IL-1*β*. ^∗^^/#^*p* < 0.05, ^∗∗^^/##^*p* < 0.01, ^∗∗∗^^/###^*p* < 0.001, and ^∗∗∗∗^^/####^*p* < 0.0001; ns: no significance. Scale bar = 10 *μ*m.

Firstly, we explored the expression changes of CGRP and its receptors, CALCRL and RAMP1, during the progression of IVDD using immunohistochemical analysis. As presented in Figure 1(a), the protein expression of CGRP and its receptors, CALCRL and RAMP 1, was increased with the progression of IVDD, with the expression of CGRP and CALCRL changing significantly (Figures 1(a) and 1(b)). IL-1*β* has been proved to be the key cytokine to promote IVDD [31]. Therefore, we established a cell model of IVDD using IL-1*β*. Human NP cells injured using IL-1*β* exhibited higher mRNA expression of CALCRL and RAMP1 (Figure 1(c)). In addition, the protein translation of CGRP receptors, CALCRL and RAMP1, in human NP cells in vitro was gradually elevated with the increasing concentration of IL-1*β* (Figure 1(d)). Taken together, experiments revealed the close relationship between the expression changes of CGRP-CALCRL/RAMP1 axis and the severity of IVDD both in vitro and in vivo.

### 3.2. Elevated Protein Expression of CGRP and Its Receptor in IVD of Aging Mice

**Figure 2:**
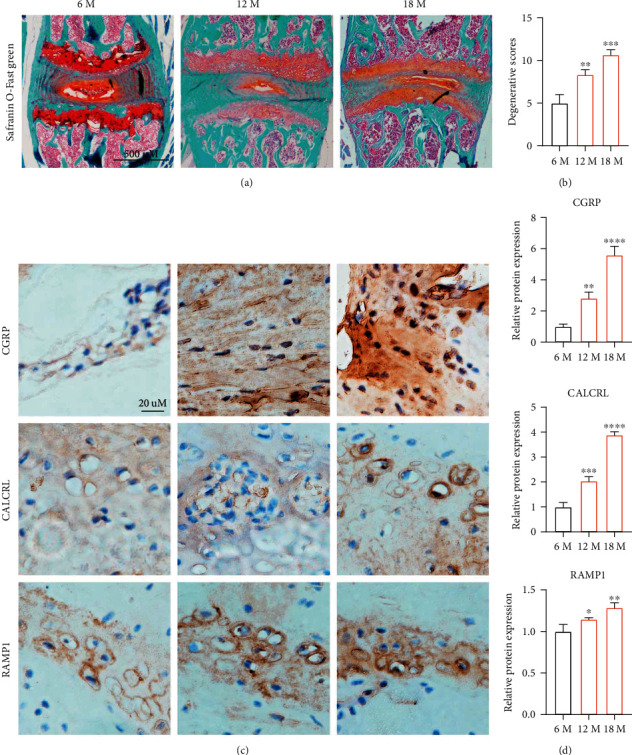
Elevated expression of CGRP and its receptor in aging mice lumbar intervertebral disc tissue. (a, b) Safranin O-fast green staining showed the shape of IVD was collapsed in aged mice at 12 months and 18 months compared with 6 months, suggesting the intervertebral disc gradually experienced degeneration with aging. (c, d) Immunohistochemical analysis revealed the gradually elevating protein expression of CGRP and its receptors, CALCRL and RAMP1, with increasing age in mice. ^∗^*p* < 0.05, ^∗∗^*p* < 0.01, ^∗∗∗^*p* < 0.001, and ^∗∗∗∗^*p* < 0.0001; ns: no significance.

To further explore the relationship between CGRP and its receptors, CALCRL and RAMP1, and age, mice with different age of months were further used. As shown in safranin O-fast green staining of Figure 2, the shape of IVD tissue was collapsed in aged mice at 12 months and 18 months compared with 6 months, suggesting the intervertebral disc gradually experienced degeneration with aging (Figures 2(a) and 2(b)). In addition, immunohistochemical analysis revealed the gradually elevated protein expression of CGRP and its receptors, CALCRL and RAMP1, with increasing age in mice (Figures 2(c) and 2(d)). As a result, CGRP and its receptors, CALCRL and RAMP1, were closely correlated with IVDD due to aging.

### 3.3. CGRP Inhibited Proliferation and Induced Apoptosis In Vitro

**Figure 3:**
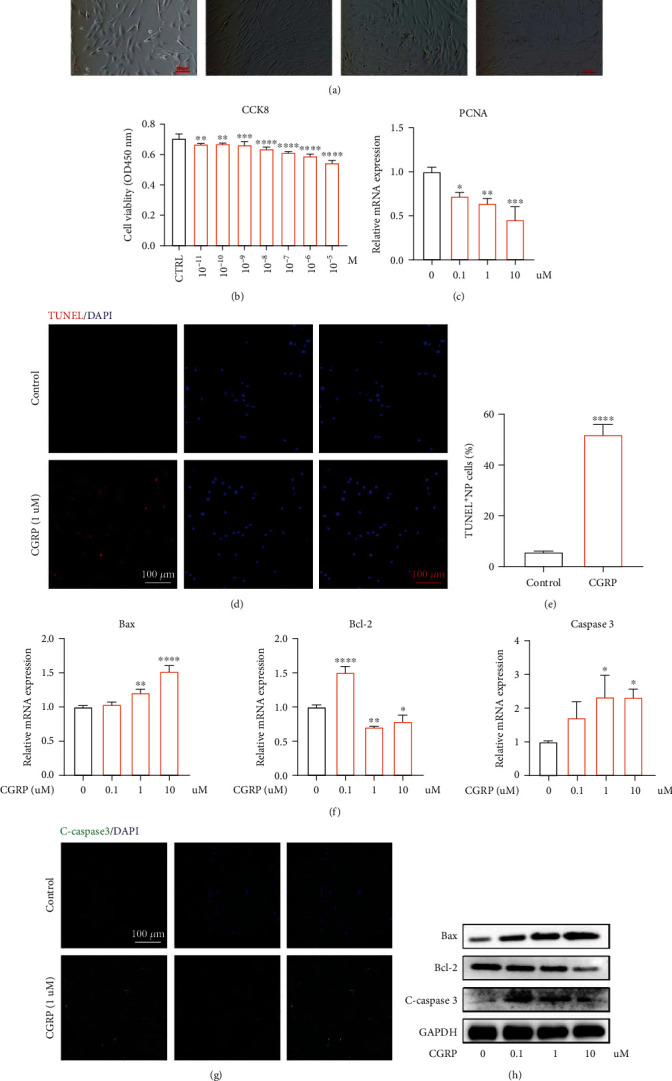
CGRP repressed cell proliferation and triggered cell apoptosis in vitro. (a) CGRP changed the cell shape in a dose-dependent manner, which exhibited a long stripe, polygon, and even collapsed shape. (b) CCK 8 analysis indicated that the NP cell viability was concentration-dependently decreased by CGRP. (c) The gene transcription of PCNA in human NP cells dose-dependently decreased after treated with CGRP. (d, e) Human NP cells treated with CGRP showed higher apoptotic percentage, indicating the proapoptotic effect of CGRP on human NP cells. (f) CGRP expedited the gene transcription of C-caspase 3 and Bax and inhibited the gene transcription of Bcl-2 with the concentration above 1 *μ*M. (g) Immunofluorescence results suggested that the protein expression of Cleaved-caspase 3 was elevated in CGRP-treated human NP cells. (h) CGRP could dose-dependently promote cell apoptosis in protein level. ^∗^*p* < 0.05, ^∗∗^*p* < 0.01, ^∗∗∗^*p* < 0.001, and ^∗∗∗∗^*p* < 0.0001; ns: no significance. Scale bar = 100 *μ*m.

Human NP cells without being stimulated by CGRP exhibited a spindle/fusiform shape. However, administration with CGRP dose-dependently changed the shape of human NP cells, which exhibited s long, polygon, and even collapsed shape (Figure 3(a)). CCK 8 analysis demonstrated that the cell viability of NP cells decreased after being treated by CGRP in a concentration-dependent manner (Figure 3(b)). Proliferating-cell nuclear antigen (PCNA) has been considered a maker for cell proliferation [32]. The mRNA expression of PCNA in human NP cells dose-dependently decreased after being treated with CGRP (Figure 3(c)). TUNEL assay showed that human NP cells treated with CGRP showed a higher apoptotic percentage (Figures 3(d) and 3(e)). Cleaved-caspase 3 and Bax have been proved to be proapoptosis markers, and Bcl-2 is the antiapoptosis marker [33]. CGRP enhanced the gene transcription of Bax and caspase 3 and inhibited the gene transcription of Bcl-2 when the concentration was above 1 *μ*M (Figure 3(f)). Immunofluorescence results suggested that the protein translation of Cleaved-caspase 3 was elevated in CGRP-stimulated human NP cells (Figure 3(g)). Furthermore, western blot analysis confirmed the results above that CGRP could dose-dependently promote apoptosis of human NP cells (Figure 3(h)). Taken together, CGRP could dose-dependently inhibit cell proliferation and triggered cell apoptosis in vitro.

### 3.4. CGRP Could Promote the Activation of Inflammation

**Figure 4:**
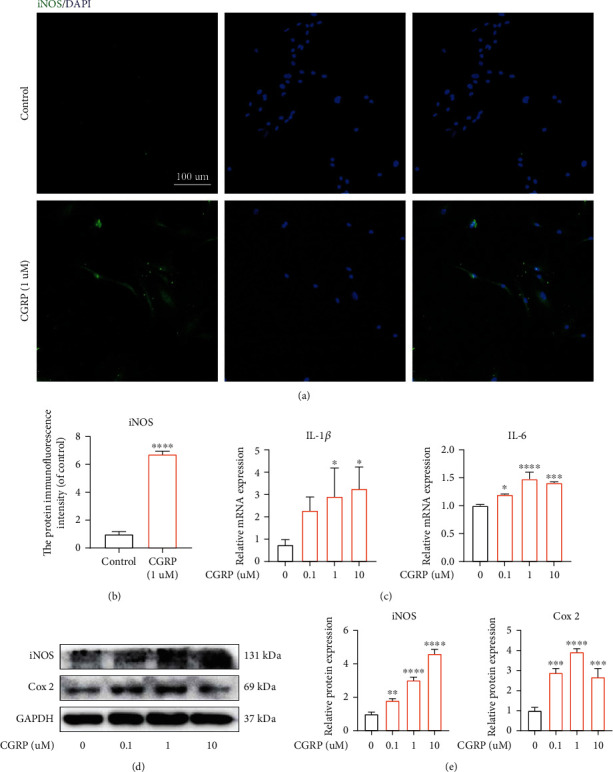
CGRP could promote the activation of inflammation. (a, b) iNos-positive cells were increased significantly in CGRP-treated human NP cells. (c) The gene transcription of inflammatory mediators, IL-1*β* and IL-6, in human NP cells was dose-dependently elevated after being stimulated with CGRP. (d, e) The protein translation of proinflammatory proteins, iNos and Cox2, was expedited by CGRP in a concentration-dependent manner. ^∗^*p* < 0.05, ^∗∗^*p* < 0.01, ^∗∗∗^*p* < 0.001, and ^∗∗∗∗^*p* < 0.0001; ns: no significance. Scale bar = 100 *μ*m.

The crucial role of inflammation played in the pathological progression of IVDD has been well identified [34]. In this present study, immunofluorescence results suggested that iNos-positive cells were increased significantly in CGRP-treated human NP cells (Figures 4(a) and 4(b)). Furthermore, the gene transcription of inflammation-related cytokines, IL-1*β* and IL-6, in human NP cells was dose-dependently elevated after being stimulated with CGRP (Figure 4(c)). Additionally, the protein translation of proinflammatory proteins, iNos and Cox2, was elevated concentration-dependently by CGRP (Figures 4(d) and 4(e)). These results above confirmed the proinflammatory effects of CGRP in human NP cells.

### 3.5. CGRP Induced ECM Degradation In Vitro

**Figure 5:**
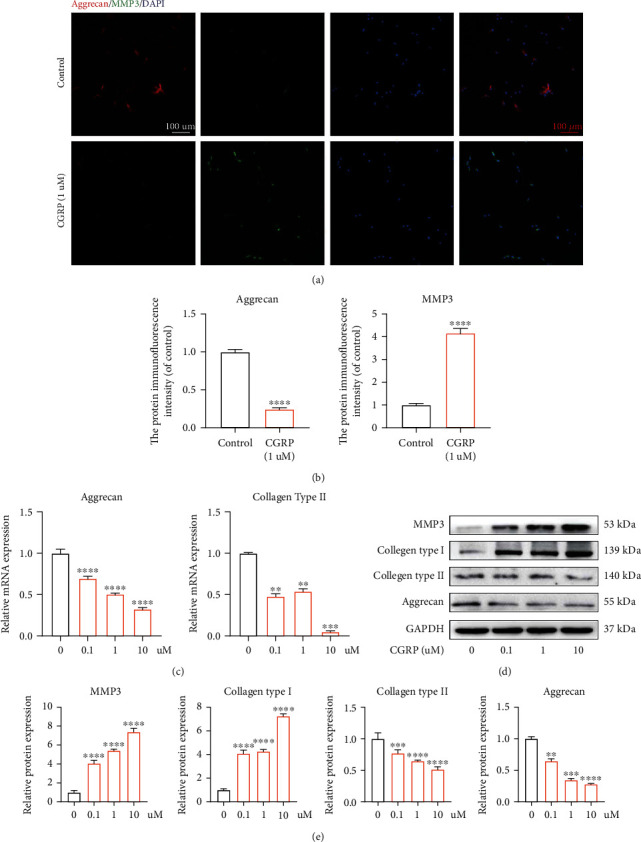
CGRP expedited ECM degradation in vitro. (a, b) Immunofluorescence results suggested higher protein translation of MMP 3 and lower protein translation of aggrecan in human NP cells with the treatment of CGRP. (c) The result of qRT-PCR analysis revealed that inhibitory effects of CGRP on mRNA transcription of aggrecan and type II collagen in NP cells were dose-dependent. (d, e) Higher protein translation of MMP 3 and lower protein translation of aggrecan in NP cells with administration of CGRP were observed according to the result of western blot analysis. ^∗^*p* < 0.05, ^∗∗^*p* < 0.01, ^∗∗∗^*p* < 0.001, and ^∗∗∗∗^*p* < 0.0001; ns: no significance. Scale bar = 100 *μ*m.

Reduced aggrecan and type II collagen and increased matrix metalloproteinase (MMP) and type I collagen within NP tissue have been proved to be major markers during the progression of IVDD [35]. Immunofluorescence results suggested higher protein translation of MMP 3 and lower protein translation of aggrecan in CGRP-stimulated human NP cells (Figures 5(a) and 5(b)). qRT-PCR analysis revealed that inhibitory effects of CGRP on gene transcription of type II collagen and aggrecan in vitro were dose-dependent (Figure 5(c)). In addition, western blot analysis showed the similar tendency (Figures 5(d) and 5(e)). Collectively, CGRP could directly trigger degenerated phenotype in human NP cells.

### 3.6. CGRP Participated in IVDD via Activating Mitogen-Activated Protein Kinases (MAPK) and Nuclear Factor-*κ*B (NF-*κ*B) Signaling Pathways

**Figure 6:**
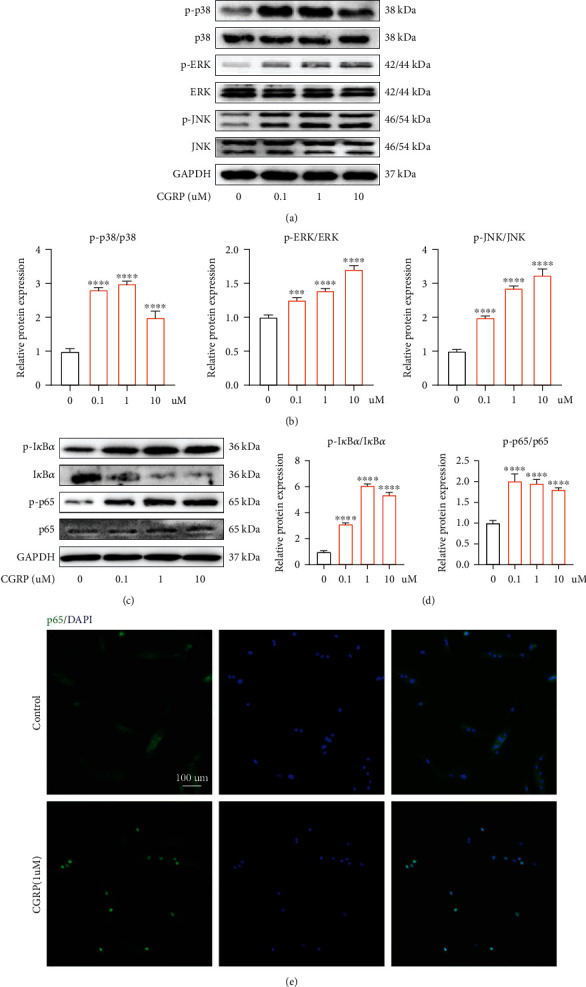
CGRP induced the activation of NF-*κ*B and MAPK signaling pathways. (a, b) CGRP dose-dependently promoted the phosphorylation of MAPK (p38, ERK, and JNK). (c, d) NF-*κ*B signaling pathway was also concentration-dependently activated by CGRP, and this activated effect was achieved through the phosphorylation of I*κ*B-*α*. (e) Immunofluorescence results suggested that the nucleus translocation of p65 was increased when human NP cells were treated with CGRP. ^∗^*p* < 0.05, ^∗∗^*p* < 0.01, ^∗∗∗^*p* < 0.001, and ^∗∗∗∗^*p* < 0.0001; ns: no significance. Scale bar = 100 *μ*m.

Pathological activation of NF-*κ*B and MAPK signaling pathway has been closely associated with IVDD, including promoting ECM degradation, cell apoptosis, and inflammatory positive feedback loop [36–38]. In this present study, western blot demonstrated that CGRP could dose-dependently enhance the phosphorylation of MAPK (p38, ERK, and JNK) (Figures 6(a) and 6(b)). Besides, NF-*κ*B signaling pathway was also concentration-dependently activated by CGRP, and this activated effect was achieved through the phosphorylation of I*κ*B-*α* (Figures 6(c) and 6(d)). Immunofluorescence results suggested that the nucleus translocation of p65 was increased when human NP cells were treated with CGRP (Figure 6(e)). These results above suggested the CGRP may exert its effects on IVDD through directly activating MAPK and NF-*κ*B signaling pathways.

### 3.7. Novel CGRP Receptor Antagonist, Rimegepant, Could Ameliorate CGRP-Mediated IVDD In Vitro

**Figure 7:**
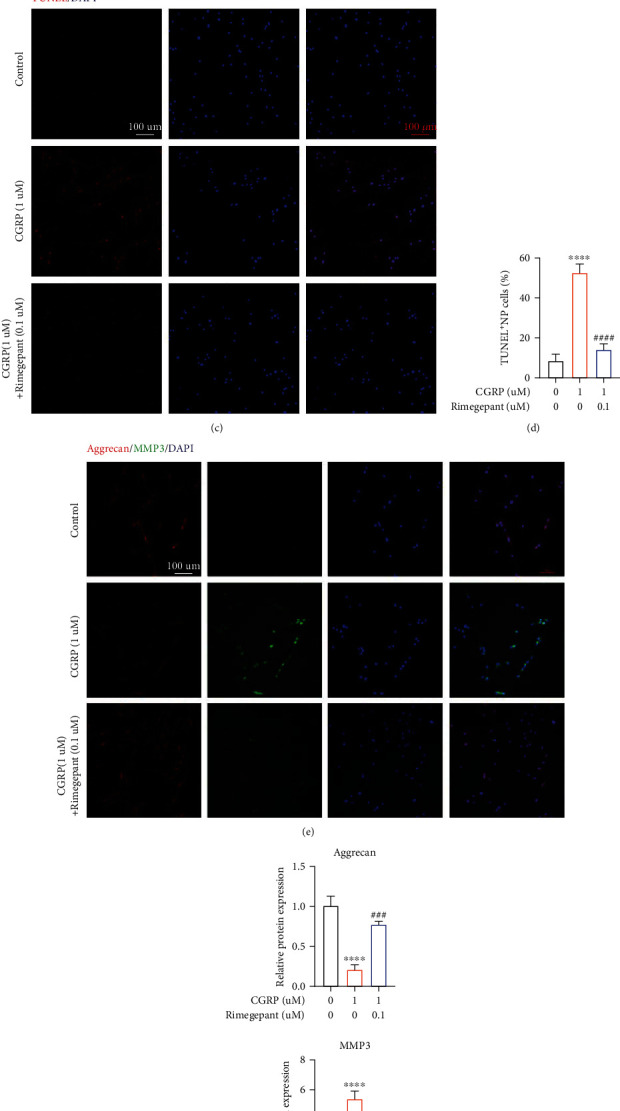
Novel CGRP receptor antagonist, Rimegepant, could ameliorate CGRP-mediated apoptosis and ECM degradation in vitro. (a) Rimegepant concentration screening was carried out using CCK 8 assay, and the results showed that Rimegepant less than 10 *μ*M is nontoxic to human NP cells. (b) Rimegepant could restore CGRP-mediated decreased cell viability in human NP cells. (c, d) TUNEL assay revealed that TUNEL-positive NP cells were increased significantly in the CGRP group, whereas human NP cells treated with CGRP plus Rimegepant showed decreased TUNEL-positive NP cells compared to the control group. (e, f) Immunofluorescence analysis suggested less protein translation of aggrecan and higher protein translation of MMP 3 in CGRP-treated human NP cells. However, Rimegepant could reverse these effects. ^∗^^/#^*p* < 0.05, ^∗∗^^/##^*p* < 0.01, ^∗∗∗^^/###^*p* < 0.001, and ^∗∗∗∗^^/####^*p* < 0.0001; ns: no significance. Scale bar = 100 *μ*m.

Rimegepant, also known as BMS-927711, is a novel small molecular compound that potently antagonizes the CGRP receptors, both CALCRL and RAMP1 [39]. Therefore, we explored whether Rimegepant had protective effects on CGRP-induced IVDD in vitro in this present study. Firstly, Rimegepant concentration screening was carried out using CCK 8 assay, and the results showed that Rimegepant less than 10 *μ*M is nontoxic to human NP cells (Figure 7(a)). In addition, Rimegepant could restore CGRP-mediated decreased cell viability in human NP cells (Figure 7(b)). TUNEL assay revealed that TUNEL-positive NP cells were increased significantly in the CGRP group, whereas human NP cells treated with CGRP plus Rimegepant showed decreased TUNEL-positive NP cells compared to the control group (Figures 7(c) and 7(d)). In terms of ECM metabolism, immunofluorescence analysis suggested less protein translation of aggrecan and higher protein translation of MMP 3 in CGRP-treated human NP cells. However, Rimegepant could reverse these effects (Figures 7(e) and 7(f)). Therefore, CGRP receptor antagonist, Rimegepant, could ameliorate CGRP-mediated cell apoptosis and ECM degradation in vitro.

### 3.8. Evaluation of the Therapeutic Effects of Rimegepant on Needle Injury-Induced IVDD In Vivo

**Figure 8:**
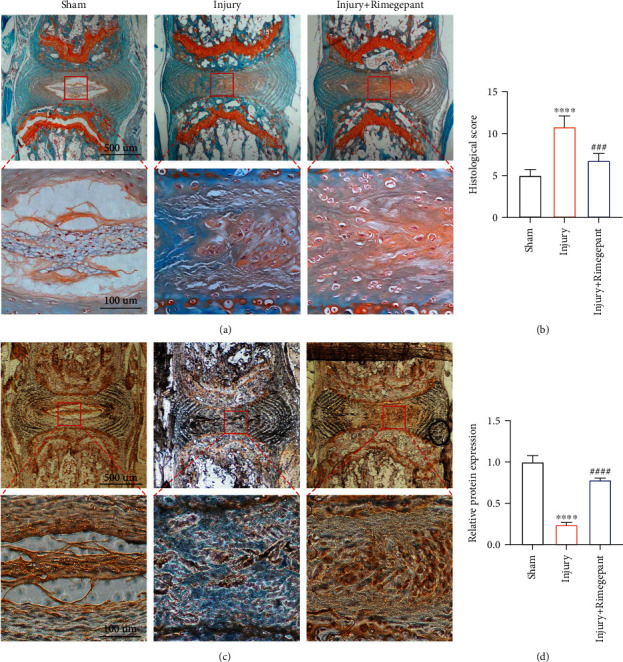
Evaluation of the therapeutic effects of Rimegepant on injury-induced IVDD in vivo. (a, b) The intervertebral disc was degenerated significantly in the injury group, whereas this effect was alleviated by Rimegepant administration. (c, d) Immunohistochemical analysis revealed that mice in the injury+Rimegepant group showed higher protein translation of type II collagen compared to those in the injury group. ^∗^^/#^*p* < 0.05, ^∗∗^^/##^*p* < 0.01, ^∗∗∗^^/###^*p* < 0.001, and ^∗∗∗∗^^/####^*p* < 0.0001; ns: no significance.

**Figure 9:**
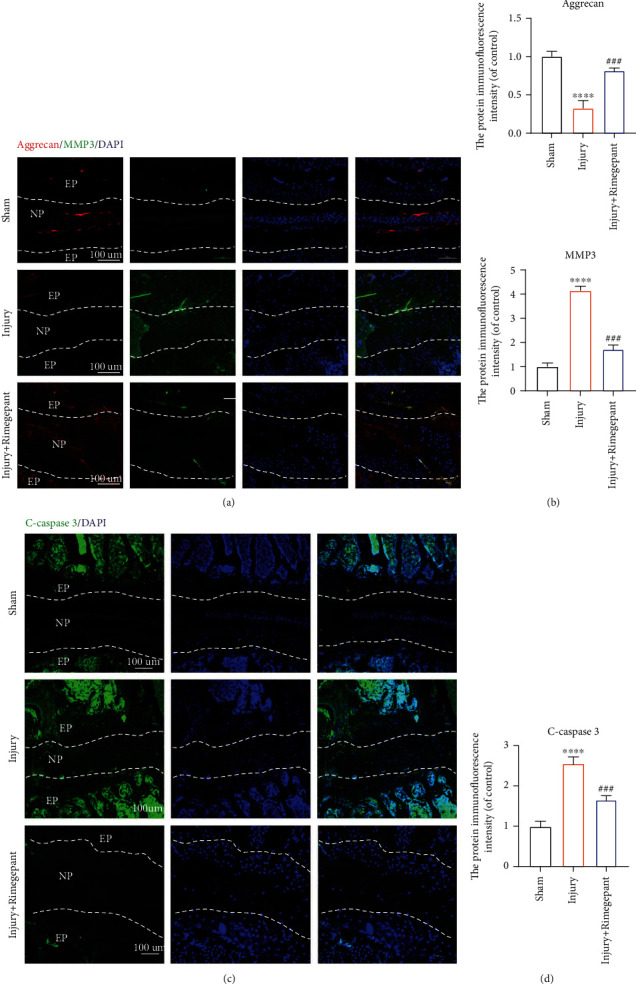
Evaluation of the therapeutic effects of Rimegepant on injury-induced IVDD in vivo. (a, b) Immunofluorescence results suggested that higher protein translation of MMP 3 and lower protein translation of aggrecan were observed in the injury group, whereas these effects were significantly restored after being treated by Rimegepant. (c, d) Cell apoptosis in vivo was also evaluated, and the expression of Cleaved-caspase 3 was enhanced in the injury group compared to the sham group, while in the injury+Rimegepant group, the protein translation of Cleaved-caspase 3 was ameliorated in comparison to the injury group. ^∗^^/#^*p* < 0.05, ^∗∗^^/##^*p* < 0.01, ^∗∗∗^^/###^*p* < 0.001, and ^∗∗∗∗^^/####^*p* < 0.0001; ns: no significance. Scale bar = 100 *μ*m.

The favorable therapeutic effects of Rimegepant on CGRP-induced IVDD in vitro were further investigated in vivo. Safranin O-fast green staining demonstrated that the intervertebral disc was degenerated significantly in the injury group in comparison to the sham group, whereas this effect was alleviated by Rimegepant (Figures 8(a) and 8(b)). Immunohistochemical analysis demonstrated that the protein translation of type II collagen was decreased in the injury group compared to the sham group. However, mice in the injury+Rimegepant group showed restored protein translation of type II collagen in comparison to those in the injury group (Figures 8(c) and 8(d)). Immunofluorescence analysis suggested higher protein content of MMP 3 and lower protein content of aggrecan in the injury group, whereas these effects were significantly restored after being treated by Rimegepant (Figures 9(a) and 9(b)). In addition, cell apoptosis in vivo was also evaluated, as suggested by increased protein expression of Cleaved-caspase 3 in the injury group versus the sham group, while in the injury+Rimegepant group, the protein expression of Cleaved-caspase 3 was ameliorated (Figures 9(c) and 9(d)). Combining the above results, we confirmed the therapeutic effects of Rimegepant in vivo, which may provide a new insight into the treatment strategy for IVDD.

## 4. Discussion

For the first time, we focused on the direct relationship between neuromodulation and IVDD, and human sample, animal model, and molecular analysis were adopted. This current study revealed that (1) degenerated or aging IVD tissue expressed a higher amount of CGRP and its receptors, CALCRL and RAMP1, which increased positively with the severity of IVDD and aging; (2) CGRP could inhibit the cell proliferation and induce cells apoptosis, inflammation, and degenerated phenotype in vitro; and (3) the therapeutic effects of CGRP receptor antagonist, Rimegepant, on IVDD were confirmed both in vitro and in vivo. Our results will bring about a novel insight into the direct role of neuromodulation in IVDD and related therapeutic attempts.

The pathological processes of IVDD are characterized as compromised cell proliferation and abnormally increased cell apoptosis. Previous studies have demonstrated that CGRP exerted inhibitory effect on cell proliferation [40, 41]. Recently, Stöckl et al. reported that CGRP increased cell apoptosis and senescence and compromised ECM metabolism in OA chondrocytes [26]. However, the exact effects of CGRP on human NP cell proliferation and apoptosis remain unclear. PCNA is an ideal marker for cell proliferation [42]. As shown in our results, the gene transcription of PCNA was dose-dependently decreased by CGRP in human NP cells. Cleaved-caspase 3, Bax, and Bcl-2 are critical markers to reflect cell apoptosis [43, 44]. CGRP elevated the amount of Cleaved-caspase 3 and Bax and reduced the amount of Bcl-2 in human NP cells, which was a potent evidence that CGRP could induce human NP cell apoptosis in vitro. Taken together, our present study for the first time confirmed the repressive effect of CGRP on proliferation and promotive effect on apoptosis in vitro.

IVDD is considered a process of chronic inflammatory response, characterized by increased immune cells and inflammatory mediators (such as iNOS, Cox2, and IL-1*β*) [45]. iNos and Cox2 have been proved to be the critical proinflammatory mediators in human degenerated NP tissue [46, 47]. Moreover, IL-1*β* and IL-6 are critical inflammatory cytokines directly exacerbating inflammation during IVDD [48]. CGRP has been reported to participate in inflammation response in multiple tissues, such as the brain, spinal cord, cardiovascular system, immune system, and bone [49–53] In addition, BIBN4096BS, a CGRP receptor antagonist, can alleviate inflammation-mediated pain in a rat model [54]. In terms of osteoarthritis, peripheral release of CGRP could contribute to acute neurogenic inflammation and inflammation-related pain [55]. Maleitzke et al. found that in collagen antibody-induced arthritis, CGRP enhanced joint inflammation [53]. Furthermore, CGRP has been reported as a biomarker to monitor the severity of knee osteoarthritis [56]. In the present study, those inflammation-related mediators, involving Cox-2, iNOS, IL-1*β*, and IL-6, were elevated dose-dependently after being treated by CGRP in vitro, suggesting the direct promotive effect of CGRP on inflammation activation in human NP cells.

Besides the cell apoptosis and inflammation activation, the remodeling of ECM is another characteristic during the development of IVDD. The normal human NP tissue is gel-like and composed predominantly of aggrecan and type II collagen, which will be decreased and substituted by type I collagen during IVDD [57]. As a result, we examined the effects of CGRP on the changes of ECM in vitro. As shown in the results, CGRP could dose-dependently decrease the protein amount of aggrecan and type II collagen and increase the amount of MMPs and type I collagen in vitro, which were deleterious for the maintenance of ECM within IVD. In fact, in osteoarthritis, CGRP was also reported to inhibit the gene expression of aggrecan and type II collagen [26]. Therefore, during IVDD, CGRP could promote degeneration phenotype of human NP cells.

IVDD has been a complex biological process, involving various signaling pathways. The activation of MAPK pathway has been reported to contribute to IVDD through various mechanisms, including ECM metabolic imbalance, cell senescence and apoptosis, oxidative stress, inflammation responses, and abnormal autophagy [58]. In addition, NF-*κ*B pathway is also indispensable for maintaining IVD homeostasis, and pathological activation of NF-*κ*B pathway correlated closely with abnormal biological processes above, such as ECM degradation [59]. Therefore, in this present study, these two pathways were examined. We found that after stimulation with CGRP, NF-*κ*B and MAPK signaling pathways were dose-dependently activated, and the dose-dependent results indicated that NF-*κ*B and MAPK signaling pathways may participate in cell apoptosis, inflammation activation, and ECM degradation during IVDD induced by CGRP.

Currently, most of the conservative treatment for IVDD, such as anti-inflammatory drugs, has been limited to symptomatic treatment and cannot reverse or alleviate the progression of IVDD. Surgical intervention is often considered the last resort, since the intraoperative risks and postoperative complications are hard to address. Therefore, it is an urgent demand to develop new drugs that are effective and safe. The result from our present study indicated that CGRP and its receptor were activated abnormally in degenerated IVD both in vitro and in vivo, and the CGRP-CALCRL/RAMP1 axis had a promotive effect on IVDD possibly via the NF-*κ*B and MAPK signaling pathway. Therefore, the CGRP receptor can be a therapeutic target for IVDD. Rimegepant, also known as BMS-927711, is a novel small molecular compound that potently antagonizes the CGRP receptor [60]. Our results indicated that Rimegepant could inhibit the adverse effects of CGRP on human NP cells in vitro. More importantly, Rimegepant could delay CGRP-induced IVDD in vivo.

Despite the inspiring results above, this present study had some limitations that should be acknowledged. First is the relatively small sample size of human NP tissues acquired. However, although the sample size was limited, various analyses, including western blot, immunohistochemical staining, and immunofluorescence staining, were adopted to minimize the bias. Moreover, the human NP cells treated with IL-1*β* exhibited consistent results. Therefore, the results and conclusion of this study are reliable. Secondly, we only applied CGRP receptor antagonist but did not directly knock down CGRP or its receptor. We believe that the result regarding the effects of CGRP on NP cells will be more confirmative if animal models with specific CGRP knockdown are applied. Thirdly, in spite of the therapeutic effects of Rimegepant that were confirmed in vitro and in vivo, the clinical use of Rimegepant for treating IVDD still has a long way to go.

## 5. Conclusion

Degenerated IVD exhibited elevated expression of CGRP and its receptor with the progression of IVDD in both human and mouse samples. In addition, CGRP could inhibit the proliferation and promote apoptosis, inflammation, and ECM degeneration through the activation of NF-*κ*B and MAPK signaling pathways in vitro. The therapeutic effects of CGRP receptor antagonist, Rimegepant, on IVDD were confirmed in vitro and in vivo. Our results will provide a brand-new perspective into the direct role of neuromodulation in IVDD and related therapeutic attempts.

## Figures and Tables

**Table 1:** Primer sequence for gene used in this present study.

Gene	Forward	Reverse
CALCRL	TCCTGCTTTAGGACCATCA	GCAGAAGAAGATTTACCACAA
RAMP1	TGCCTCACCAGTTCCAG	CAGCTTCTCCGCCATGTG
PCNA	GCCTGACAAATGCTTGCT	GCGGGAGGAGGAAAGT
BAX	TGCGTCCACCAAGAAGC	TCCAGTTCGTCCCCGAT
BCL-2	GCGGATTGACATTTCTGTG	CATAAGGCAACGATCCCA
Caspase 3	CAGTGATGCTGTGCTATGAAT	CAGATGCCTAAGTTCTTCCAC
Aggrecan	CTATACCCCAGTGGGCACAT	GGCACTTCAGTTGCAGAAGG
Type II collagen	CCAGATGACCTTCCTACGCC	GGCACTTCAGTTGCAGAAGG
IL-1*β*	TTGAGTCTGCCCAGTTCC	TTTCTGCTTGAGAGGTGCT
IL-6	CAATGAGGAGACTTGCCTGG	GCACAGCTCTGGCTTGTTCC
GAPDH	TGACCACAGTCCATGCCATC	GACGGACACATTGGGGGTAG

## Data Availability

Data in this study would be available if required.
